# 6-Gingerol Normalizes the Expression of Biomarkers Related to Hypertension via PPAR*δ* in HUVECs, HEK293, and Differentiated 3T3-L1 Cells

**DOI:** 10.1155/2018/6485064

**Published:** 2018-12-16

**Authors:** Yong-Jik Lee, Yoo-Na Jang, Yoon-Mi Han, Hyun-Min Kim, Hong Seog Seo

**Affiliations:** ^1^Cardiovascular Center, Korea University Guro Hospital, 148 Gurodong-ro, Guro-gu, Seoul 08308, Republic of Korea; ^2^Department of Medical Science, Korea University College of Medicine (BK21 Plus KUMS Graduate Program), Main Building 6F Room 655. 73, Inchon-ro (Anam-dong 5-ga), Seongbuk-gu, Seoul 136- 705, Republic of Korea

## Abstract

Hypertension is a disease with a high prevalence and high mortality rates worldwide. In addition, various factors, such as genetic predisposition, lifestyle factors, and the abnormality of organs related to blood pressure, are involved in the development of hypertension. However, at present, there are few available drugs for hypertension that do not induce side effects. Although the therapeutic effects of ginger on hypertension are well established, the precise mechanism has not been elucidated. Therefore, this study was designed to evaluate the antihypertensive mechanism of 6-gingerol, one of the main ingredients of ginger, and to assist in the development of new drugs for hypertension without side effects. The antihypertensive effects and mechanism of 6-gingerol were identified through reverse transcription polymerase chain reaction (RT-PCR), western blotting, and immunocytochemical staining for biomarkers involved in hypertension in human umbilical vein endothelial cells (HUVECs), human embryonal kidney cells (HEK293 cells), and mouse preadipocytes (3T3-L1 cells). The lipid accumulation in differentiated 3T3-L1 cells was evaluated by using Oil Red O staining. 6- Gingerol increased the level of phosphorylated endothelial nitric oxide synthase (eNOS) protein but decreased that of vascular cell adhesion protein 1 (VCAM1) and tumor necrosis factor alpha (TNF*α*) in HUVECs. In HEK293 cells, the expression of the epithelial sodium channel (ENaC) protein was reduced by 6-gingerol. Lipid accumulation was attenuated by 6-gingerol treatment in differentiated 3T3-L1 cells. These effects were regulated via peroxisome proliferator-activated receptor delta (PPAR*δ*). 6-Gingerol ameliorated the expression of biomarkers involved in the development of hypertension through PPAR*δ* in HUVECs, HEK293, and differentiated 3T3-L1 cells.

## 1. Introduction

Blood pressure is defined as the force of blood pushing against the walls of arteries as it is pumped by the heart; hypertension is the state in which blood pressure is persistently increased [1]. In general, hypertension has various causes, such as lifestyle, sex, age, obesity, genetic problems, and kidney dysfunction. Globally, approximately 40% of adults aged 25 years and older were diagnosed as having hypertension in 2008 [2], and the complications of hypertension are responsible for the deaths of approximately 9.4 million people per year [3]. Therefore, hypertension and its complications lower quality of life and represent a huge burden on societies and nations. Although many antihypertensive drugs have been developed and marketed, there are nearly as many side effects as there are antihypertension drugs. The need to find and develop new and safer drugs to treat hypertension is, therefore, a priority.

Hypertension can cause atherosclerosis (a type of arteriosclerosis); however, atherosclerosis itself also induces hypertension. Hypertension induces arterial damage and the formation of atherosclerotic plaques inside arteries. Atherosclerotic plaques, which consist of cholesterol, fat components, calcium, fibrin, and foam cells, induce the hardening and narrowing of arteries and may lead to serious cerebrocardiovascular diseases or death [4]. High blood pressure and kidney dysfunction have reciprocal effects: high blood pressure induces damage to renal vessels via vessel stretch; in contrast, kidney impairment leads to fluid accumulation in vessels, improper excretion, hyperaldosteronism, and elevated sodium reabsorption, and these defects then raise blood pressure. Thus, abnormalities in vessels and the kidneys are reflective of the hypertensive state of the body. In addition to abnormalities related to vascular tissue and the kidneys, obesity is a serious risk factor for the onset of hypertension [5, 6]. Hence, hypertension and obesity exert adverse effects that mutually cause the other.

Ginger (*Zingiber officinale*) is one of the most commonly consumed spices in the world [7] and has been considered an important medicine in human history. According to Dongui Bogam (the Korean Traditional Medical Encyclopedia), ginger warms the body, strengthens the gastrointestinal system, and reduces vomiting. [6]-Gingerol (1-[4′-hydroxy-3′-methoxyphenyl]-5-hydroxy-3-decanone) is the primary pungent ingredient responsible for the various therapeutic effects of ginger. In southern Asia, it is known that ginger has effects on cardiovascular diseases [8, 9]. Recent studies on the roles of gingerol extracts and gingerol, mainly in metabolic disorders, have drawn the following conclusions: gingerol extract decreases diet-induced obesity and increases endurance capacity through an increase in fat utilization via PPAR*δ* signaling [10], and 6-gingerol mediates blood pressure effects through the inhibition of angiotensin II type 1 receptor (AT1R) activation [11], enhances glucose uptake via AMPK in differentiated L6 rat skeletal myocytes [12], and inhibits inflammation via AMPK activation in colitis [13]. Although a wealth of research has been conducted on the effects of gingerol in various disease conditions, there are relatively few studies on the role of gingerol in hypertension.

The regulation of blood pressure is mainly related to the modulation of vascular constriction, reabsorption of sodium ions in the kidney, and lipid metabolism disorders. Therefore, in this study, to investigate the effects of 6-gingerol on hypertension and the underlying mechanisms, we measured the protein expression of biomarkers related to blood pressure in HEK293 human embryo kidney cells exposed to high-salt conditions and human umbilical vein endothelial cells (HUVECs) maintained in high cholesterol and high fatty acid conditions. In addition, the effect and mechanism of 6-gingerol against lipid accumulation were investigated in differentiated 3T3-L1 cells.

## 2. Materials and Methods

### 2.1. Materials

The HEK293 human embryonic kidney cell line, 3T3-L1 mouse embryonic fibroblasts (preadipocytes), and the CPAE cow pulmonary artery endothelial cell line were purchased from the Korean Cell Line Bank (Seoul, Korea). HUVECs were donated by Dr. Geum-Joon Cho (Department of Obstetrics and Gynecology, Korea University, Guro Hospital). The reagents for cell culture, including Dulbecco's Modified Eagle Medium (DMEM), fetal bovine serum (FBS), fetal calf serum (FCS), and antibiotic-antimycotic solution (AA), were purchased from WELGENE Inc. (Daegu, Korea). The EGM™-2 BulletKit™, an endothelial cell growth medium kit, was acquired from LONZA (Basel, Switzerland). Protein extraction solution and prestained protein markers were obtained from Intron Biotechnology (Seongnam-si, Gyeonggi-do, Korea). Sodium chloride (NaCl), 6- gingerol, 3-(((2-Methoxy-4-(phenylamino)phenyl)amino]sulfonyl)-2-thiophenecarboxylic acid methyl ester (a PPAR*δ* antagonist, also called GSK0660), dorsomorphin (an AMPK inhibitor, also called compound C), and Oil Red O reagent were purchased from Sigma-Aldrich (St. Louis, MO, USA). Kodak GBX developer and fixer reagents were purchased from Carestream Health, Inc. (Rochester, NY, USA). Primary antibodies against *β*-actin and vascular cell adhesion molecule 1 (VCAM1) and appropriate secondary antibodies were procured from Santa Cruz Biotechnology, Inc. (Dallas, TX, USA). Primary antibodies against endothelial nitric oxide synthase (eNOS), phosphorylated eNOS (p-eNOS), 5′-adenosine monophosphate kinase (AMPK), and phosphorylated AMPK (p-AMPK) were purchased from Cell Signaling Technology, Inc. (Danvers, MA, USA). Primary antibodies for the detection of peroxisome proliferator-activated receptor delta (PPAR*δ*) and peroxisome proliferator-activated receptor gamma coactivator 1-alpha (PGC-1*α*) were supplied by Abcam (Cambridge, UK). The primary antibodies for the detection of antitumor necrosis factor alpha (TNF*α*) and epithelial sodium channel (ENaC) were purchased from Novus (Littleton, CO, USA). TRIzol® reagent was purchased from Invitrogen (Grand Island, NY, USA). A Power cDNA Synthesis kit, PCR Premix, and DNA ladder (100 base pair) were obtained from Intron Biotechnology (Seongnam-si, Gyeonggi-do, Korea).

### 2.2. Cell Culture

HEK293 cells were cultured in DMEM containing 10% FBS and 1% AA at 37°C in a 5% CO_2_ incubator. Fresh medium was supplied every 48–72 h. For experiments, cells between passage 59 and 63 were plated at a density of 5×10^5^ cells per well (9.6 cm^2^) in 6-well culture plates. The cells were cultured for 24 to 48 h at 37°C in a 5% CO_2_ incubator, and the medium was changed to DMEM containing 1% FBS. Thereafter, HEK293 cells were simultaneously treated with NaCl (54.75 mmol), 6-gingerol (50 *μ*mol), and GSK0660 (50 *μ*mol) for 24 h. HUVECs were cultured in endothelial cell growth medium, with fresh medium supplied every 48–72 h. For experiments, HUVECs between passage 7 and 12 were plated in 6-well culture plates at a density of 1×10^6^ cells per well and cultured for 24–48 h at 37°C in a 5% CO_2_ incubator. After the medium was exchanged for new medium, HUVECs were simultaneously treated with cholesterol (0.1 mmol), palmitate (0.1 mmol), 6-gingerol (50 *μ*mol), and GSK0660 (50 *μ*mol) for 24 h. CPAE cells were cultured in DMEM containing 10% FBS and 1% AA at 37°C in a 5% CO_2_ incubator. Fresh medium was supplied every 48–72 h. For experiments, CPAE cells between passage 35 and 40 were plated in 6-well culture plates at a density of 1×10^6^ cells per well and cultured for 24–48 h at 37°C in a 5% CO_2_ incubator. After the medium was exchanged for DMEM containing 1% FBS, CPAE cells were simultaneously treated with cholesterol (0.1 mmol) and 6-gingerol (50 *μ*mol) for 24 h.

3T3-L1 cells between passage 9 and 18 were plated in 24-well (1.9 cm^2^/well) or 6-well culture plates at a density of 5×10^4^ or 2×10^5^ cells per well, respectively, in DMEM containing 10% calf serum and 1% AA solution. When 3T3-L1 cells reached confluence, differentiation medium was applied to cells in addition to 6-gingerol (50 *μ*mol) and a PPAR*δ* antagonist (50 *μ*mol). The differentiation medium contained 0.0125 *μ*mol/mL dexamethasone, 12.5 *μ*mol/mL 3-isobutyl-1-methylxanthine, 10 *μ*g/mL insulin, and 10% FBS. After differentiation for 2 days, the medium was replaced with insulin medium (containing 10 *μ*g/mL insulin and 10% FBS). After incubation in insulin medium for 2–4 days, the medium was exchanged for maintenance medium, which contained only 10% FBS.

### 2.3. Semiquantitative Reverse Transcription Polymerase Chain Reaction (RT-PCR)

Total RNA was extracted using TRIzol® reagent according to the manufacturer's instructions. Complementary DNA was synthesized from total RNA using the Power cDNA Synthesis kit, and polymerase chain reactions for human PPAR*δ*, human AT1R, human TNF*α*, human *β*-actin, mouse PPAR*δ*, mouse PPAR*γ*, mouse fatty acid synthase (FAS), mouse *β*-actin, bovine PPAR*δ*, bovine monocyte chemoattractant protein-1 (MCP-1), and bovine glyceraldehyde 3-phosphate dehydrogenase (GAPDH) were performed using a PCR Premix kit. The primer sequences used were as follows: forward 5'-aaggccttctccaagcacat-3' and reverse, 5'-aagacgtgcacgctgatctc-3' for human PPAR*δ* (product size- 239 base pairs); forward 5'-tcccaaaattcaacccttcc-3' and reverse, 5'- gtggggaatccaggaaaaga-3' for human AT1R (product size- 216 base pairs); forward 5'- ccctcaacctcttctggctc-3' and reverse, 5'-agctgtaggccccagtgagt-3' for human TNF*α* (product size- 163 base pairs); forward 5'- gcttggtcacttcgtggcta-3' and reverse, 5'-caaaccgcttccaactcaaa-3' for human *β*- actin (product size- 522 base pairs); forward 5'-ggcagagttgctagggttcc-3' and reverse, 5'- caaggaacaccccaagacct-3' for mouse PPAR*δ* (product size- 212 base pairs); forward 5'- agccgtgcaagagatcacag-3' and reverse, 5'- aggcttttgaggaactccc-3' for mouse PPAR*γ* (product size- 147 base pairs); forward 5'- gaaacctgacggcatcattg-3' and reverse, 5'-cggtgtcctcagagttgtgg-3' for mouse FAS (product size- 281 base pairs); forward 5'-ctaggcaccagggtgtgatg-3' and reverse, 5'- ctacgtacatggctggggtg-3' for mouse *β*-actin (product size- 281 base pairs); forward 5'- catcattctgtgcggagacc-3' and reverse, 5'-gcttggggaagaggtactgc-3' for bovine PPAR*δ* (product size- 134 base pairs); forward 5'-cctcctgtgcctgctactca-3' and reverse, 5'- gtcctggacccatttctgct-3' for bovine MCP-1 (product size- 238 base pairs); forward 5'- ccgttcgacagatagccgta-3' and reverse, 5'- aagatggtgatggcctttcc-3' for bovine GAPDH (product size- 280 base pairs). The reaction mixture containing cDNA was preheated for 5 min at 95°C as an initial denaturation step. The polymerase chain reaction consisted of a denaturation step for 20 s at 95°C, an annealing step for 10 s at 55°C, an extension step for 30 s at 72°C, and a final extension step for 3 min at 72°C.

### 2.4. Western Blot Analysis

First, the cells were homogenized in protein extraction solution, and the protein concentration of the cell extracts was estimated using the Bradford method. For each sample, 10 *μ*g of the extracted proteins were loaded onto 10% sodium dodecyl sulfate-polyacrylamide gel electrophoresis (SDS- PAGE) gels. The proteins were transferred to nitrocellulose membranes using electroblotting for 90 min at 100 V, and nonspecific binding to the membranes was blocked by overnight incubation of the membrane in 5% skim milk solution. After three 10-min washes in Tris-buffered saline containing 0.05% Tween 20 (TBS-T), the membranes were incubated with solutions of primary antibodies at room temperature (25°C) for 2 h. The following primary antibodies were used at 1:1000 dilution: PPAR*δ*, AMPK, p-AMPK (at Thr172), eNOS, p-eNOS (at Ser1177), PGC-1*α*, and *β*-actin. After three more 10-min washes in TBS-T, the membranes were incubated with horseradish peroxidase-conjugated secondary antibodies at room temperature for 1 h. The following dilutions were used for the secondary antibodies: 1:5000 for anti-rabbit IgG antibodies for PPAR*δ*, AMPK, p-AMPK, eNOS, p-eNOS, and PGC-1*α* and 1:5000 for anti-mouse IgG antibody for *β*-actin. Subsequently, the membranes were washed three times in TBS-T for 10 min, once in TBS for 10 min, and then treated with a chemiluminescent substrate and enhancer solutions. The images were obtained manually using developer and fixer reagents, and the results were analyzed using Image J software.

### 2.5. Immunocytochemistry

Cells were fixed in a chamber slide by the application of ice-cold methanol for 15 min. The intrinsic peroxidase activity in cells was eliminated by treatment with PBS containing 0.3% H_2_O_2_ and 0.3% normal serum. After a 5-min wash in PBS, the cells were incubated for 10 min in PBS containing 0.25% Triton X-100 and washed again in PBS for 5 min. The cells were then incubated in normal blocking serum (diluted 1:100 in PBS) for 20 min. After removal of the blocking serum, the cells were incubated for 1 h with the primary antibody solution, washed for 5 min, and incubated with the secondary antibody solution for 30 min. After a 5-min wash in PBS, VECTASTAIN® ABC reagent was added to the cells, which were incubated for 30 min and washed again. Subsequently, 3,3′-diaminobenzidine (DAB) substrate solution was added, and incubation continued until the expected color change had occurred. After three washes with PBS, the cells were counterstained with hematoxylin and washed with distilled water. The cells in the chamber slide were dried, covered with glass, and observed using an optical microscope. The image density was repeatedly estimated using Image J software during every experiment as follows. The image density was measured after designating a defined area in the figure (the area size was the same throughout all immunocytochemistry images). Next, the same unit of area was specified in other parts of the figure and their densities measured. The quantification was randomly repeated ten times per one image. The quantification work was repeated every time the immunocytochemistry experiment was performed. Finally, the numerical density data obtained through repeated experiments were statistically analyzed using Prism software.

### 2.6. Oil Red O Staining

Cells were seeded in 24-well culture plates, fixed in 4% formaldehyde solution for 30 min, washed with PBS for 5 min and then stained with Oil Red O solution for 1 h. After a 30 s wash in 40% isopropyl alcohol, the cells were washed twice in PBS for 5 min, observed, and photographed using an optical microscope. Absolute isopropyl alcohol (1 mL) was added to each well, after which the eluted Oil Red O was quantified through measurement of the absorption at 530 nm using a Spectramax Plus 384-well microplate reader (Molecular Devices LLC., Sunnyvale, CA, USA).

### 2.7. Oxygen Consumption Rate Analysis

Oxygen consumption rate (OCR) analysis in 3T3-L1 cells was estimated using the Seahorse XFp system (Agilent, Santa Clara, CA, USA) according to the manufacturer's protocol. Cells were plated at 10,000 cells per well, and after the settlement of cells, 6-gingerol was added to media, and the cells were incubated overnight in a 37°C, 5% CO_2_ incubator. A sensor cartridge+utility plate containing calibrant was incubated overnight in a CO_2_-free incubator at 37°C. On the day of the analysis, assay media were prepared similar to culture media (25 mM glucose and 4 mM L-glutamine), and the pH was adjusted to 7.4. The XFp miniplate was washed twice with assay media and assay media (a final volume of 180 *μ*l) were added to cells. Then, the XFp miniplate was allowed to equilibrate in a CO_2_-free incubator at 37°C for 60 min prior to assay initiation. Oligomycin, carbonyl cyanide-4-(trifluoromethoxy) phenylhydrazone (FCCP), and antimycin A/rotenone were separately injected in each drug port in the sensor cartridge+utility plate and incubated in a CO_2_-free incubator for 10 min. In the case of differentiated 3T3-L1 cells, the cells were treated with 6-gingerol for 24 h on the last day of cell differentiation, and then the OCR was measured.

### 2.8. Statistics

The data are presented as the mean ± SEM (Standard Error of Measures). Statistically significant differences between two groups were calculated using Student's t-test, and one-way ANOVA was used to examine the statistical differences among more than three groups. Values of p<0.05 were considered to indicate statistically significant differences.

## 3. Results

### 3.1. 6-Gingerol Ameliorates mRNA Levels of Biomarkers Related to Hypertension in HUVECs, HEK293 Cells, and Differentiated 3T3-L1 Cells under Pathological Conditions

**Figure 1:**
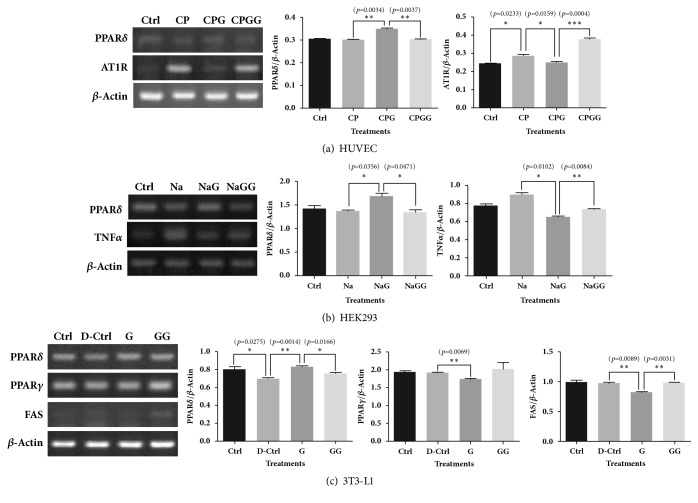
**The effects of 6-gingerol on mRNA biomarkers related to hypertension in HUVECs, HEK293 cells, and differentiated 3T3-L1 cells under pathological conditions.** (a, b, c) 6-Gingerol increased the mRNA level of PPAR*δ* compared with disease controls in HUVECs, HEK293, and differentiated 3T3-L1 cells. (b) Treatment with 6-gingerol decreased the mRNA level of AT1R elevated by cholesterol and palmitate treatment in HUVECs and downregulated the TNF*α* mRNA levels elevated by NaCl treatment in HEK293 cells. (c) In differentiated 3T3-L1 cells, 6- gingerol decreased the levels of PPAR*γ* and FAS. All effects of 6-gingerol in the three cell types were offset by the PPAR*δ* antagonist, GSK0660. The results are expressed as means ± SEM. Values were statistically analyzed using unpaired* t*-tests. All experiments were repeated three or more times. The statistical significance between the two experimental groups was marked as *∗ p *< 0.05, *∗∗ p *< 0.01, and *∗∗∗ p *< 0.001. Meaning of indications: Ctrl is an untreated control group, CP indicates a cholesterol and palmitate-treated group, CPG indicates a cholesterol, palmitate, and 6-gingerol treated group, and CPGG indicates a cholesterol, palmitate, 6-gingerol, and GSK0660 treated group; Na indicates an NaCl treated group, NaG indicates an NaCl and 6-gingerol treated group, and NaGG indicates an NaCl, 6-gingerol, and GSK0660 treated group; D-Ctrl indicates a differentiation control group without treatment, G indicates a 6-gingerol treated group during differentiation, and GG indicates a 6-gingerol and GSK0660 treated group during differentiation.

6-Gingerol increased the mRNA level of PPAR*δ* compared with the disease control group of HUVECs, HEK293 cells, and differentiated 3T3-L1 cells. Treatment with 6-gingerol decreased the level of AT1R (an essential factor in vasoconstriction) mRNA increased by cholesterol and palmitate treatment in HUVECs (Figure 1(a)) and downregulated the increased TNF*α* mRNA levels induced by NaCl treatment in HEK293 cells (Figure 1(b)). Furthermore, 6-gingerol decreased the levels of PPAR*γ* and FAS in differentiated 3T3-L1 cells (Figure 1(c)). All the effects of 6-gingerol in HUVECs, HEK293 cells, and differentiated 3T3-L1 cells were reversed by the PPAR*δ* antagonist, GSK0660 (Figure 1).

### 3.2. 6-Gingerol Increases the Protein Levels for PPAR*δ*, p-AMPK, PGC-1*α*, and p-eNOS in HUVECs Treated with High Concentrations of Cholesterol and Palmitate, and Its Function Was Dependent on* PPARδ*

In vascular endothelial cells, eNOS catalyzes the reaction producing nitric oxide (NO), which is a key regulator of blood pressure. The activation of eNOS is induced via the phosphorylation of a specific residue [14], and the regulation of eNOS is generally related to PPAR*δ*, AMPK, and PGC-1*α*. Therefore, we evaluated the protein expression of PPAR*δ*, AMPK, PGC-1*α*, and eNOS in vascular endothelial cells treated with palmitate and cholesterol at final concentrations of 50 *μ*mole.

**Figure 2:**
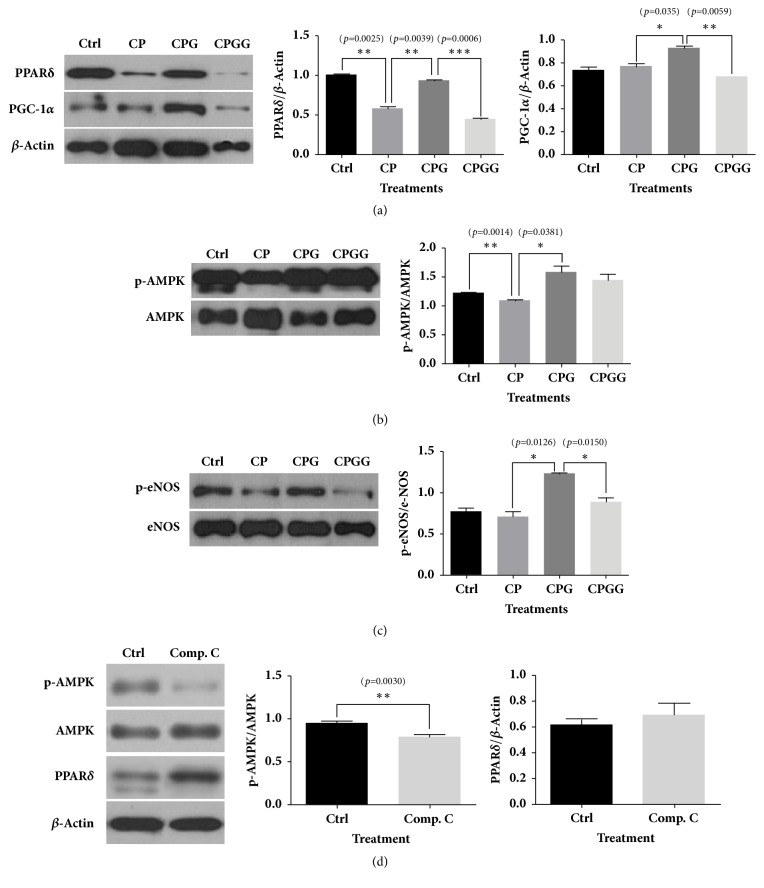
**Western blot analyses for PPAR** **δ** **, AMPK, and eNOS in HUVECs treated with cholesterol, palmitate, 6-gingerol, GSK0660, and compound C.** (a, b, c) 6-Gingerol ameliorated the levels of PPAR*δ*, AMPK, and eNOS abnormally expressed in HUVECs treated with cholesterol and palmitate. The effects of 6-gingerol were reversed by a PPAR*δ* antagonist. (d) The AMPK antagonist, compound C, did not affect the protein level of PPAR in HUVECs. The results are expressed as the means ± SEM (n=3). Values were statistically analyzed using unpaired* t*-tests. All experiments were repeated three or more times. The statistical significance between the two experimental groups was marked as *∗ p *< 0.05, *∗∗ p *< 0.01, and *∗∗∗ p *< 0.001. Meaning of indications: Ctrl indicates an untreated normal control group, CP indicates a cholesterol and palmitate-treated group, CPG indicates a cholesterol, palmitate, and 6-gingerol treated group, and CPGG indicates a cholesterol, palmitate, 6-gingerol, and GSK0660 treated group.

6-Gingerol increased the protein expression of PPAR*δ* and PGC-1*α* following cholesterol and palmitate treatment; however, this expression was decreased by GSK0660 treatment (Figure 2(a)). p-AMPK expression, which was reduced by cholesterol and palmitate treatment alone, was elevated following 6-gingerol treatment and decreased by GSK0660 treatment (Figure 2(b)). Although the expression of p-eNOS was higher in the cholesterol and palmitate-treated group than in the control group, a more significant increase in expression was observed after 6-gingerol treatment. However, GSK0660 suppressed the increased p-eNOS expression induced by 6-gingerol (Figure 2(c)). Compound C, an AMPK antagonist, decreased the level of p-AMPK without affecting PPAR*δ* in HUVECs (Figure 2(d)).

### 3.3. 6-Gingerol Decreased TNF*α* and VCAM1 Protein Expression in HUVECs Treated with High Concentrations of Cholesterol and Palmitate

**Figure 3:**
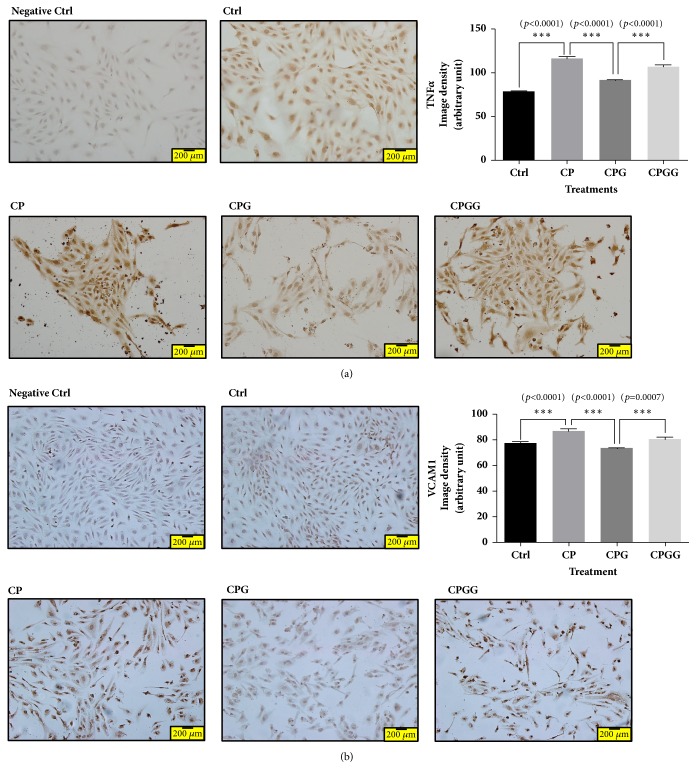
**Immunocytochemistry for TNF** **α** **and VCAM1 in HUVECs treated with cholesterol, palmitate, 6-gingerol, and GSK0660.** The increased expression of TNF*α* (a) and VCAM1 (b) proteins by cholesterol and palmitate treatment was ameliorated by 6-gingerol treatment in HUVECs. The effects of 6-gingerol were reversed by a PPAR*δ* antagonist. Magnification is 200 times. All experiments were repeated three or more times. The statistical significance between the two experimental groups was marked as *∗∗∗ p *< 0.001. Meaning of indications: Ctrl indicates an untreated normal control group, CP indicates a cholesterol and palmitate-treated group, CPG indicates a cholesterol, palmitate, and 6-gingerol treated group, and CPGG indicates a cholesterol, palmitate, 6-gingerol, and GSK0660 treated group.

Inflammation is involved in the development of atherosclerosis, as well as abnormalities of the vascular wall; consequently, it induces vascular constriction [15]. TNF*α* is a representative inflammatory cytokine that participates in the development of arteriosclerosis [16]. Furthermore, TNF*α* increases the expression of cell adhesion molecules, such as VCAM1, which participate in the occurrence of atherosclerotic plaques [17]. 6-Gingerol treatment decreased TNF*α* and VCAM1 expression, which was increased by cholesterol and palmitate treatment. However, GSK0660 treatment offset the suppressive effect of 6-gingerol on TNF*α* and VCAM1 (Figure 3).

### 3.4. ENaC Protein Expression Was Increased by NaCl Treatment but Decreased by 6-Gingerol Treatment in HEK293 Cells

The kidneys are a component of the renin-angiotensin-aldosterone system that regulates blood pressure and body fluid homeostasis. In particular, ENaC in the kidney has an essential function in blood pressure regulation [18]. Therefore, the evaluation of ENaC expression in kidney cells is an important tool to help identify the mechanism of action of 6-gingerol in hypertensive conditions.

**Figure 4:**
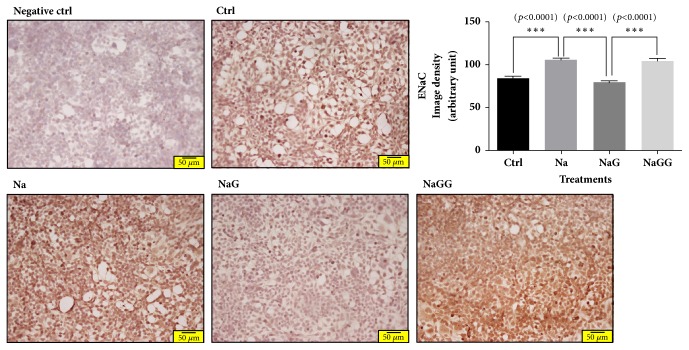
**Immunocytochemistry for ENaC in HEK293 cells treated with NaCl, 6-gingerol, and GSK0660.** In HEK293 cells, the abnormal ENaC protein expression induced by NaCl treatment was normalized by 6-gingerol treatment. The effect of 6-gingerol was reversed by pretreatment with a PPAR*δ* antagonist. Magnification is 200 times. All experiments were repeated three or more times. The statistical significance between the two experimental groups was marked as *∗∗∗ p *< 0.001. Meaning of indications: Ctrl indicates an untreated normal control group, Na indicates an NaCl treated group, NaG indicates an NaCl and 6-gingerol treated group, and NaGG indicates an NaCl, 6-gingerol, and GSK0660 treated group.

The increase in ENaC expression after NaCl treatment was suppressed to the level in control cells by treatment with 6-gingerol, and the effect of 6-gingerol was reversed by the PPAR*δ* antagonist, GSK0660 (Figure 4).

### 3.5. 6-Gingerol Increased PPAR*δ* and p-AMPK Protein Levels in HEK293 Cells Treated with High NaCl Concentrations, and Its Function Was Dependent on PPAR*δ*

In animals fed with a high-salt diet, p-AMPK expression was reduced; however, treatment with 5-aminoimidazole-4-carboxamide 1-*β*-D-ribofuranoside (AICAR, an AMPK agonist) increased AMPK activity. Moreover, the activation of AMPK induced renal tubular sodium reabsorption [19].

As AMPK regulates the reabsorption of sodium ions in kidney tubules, we investigated AMPK protein expression in HEK293 cells treated with high NaCl concentrations.

**Figure 5:**
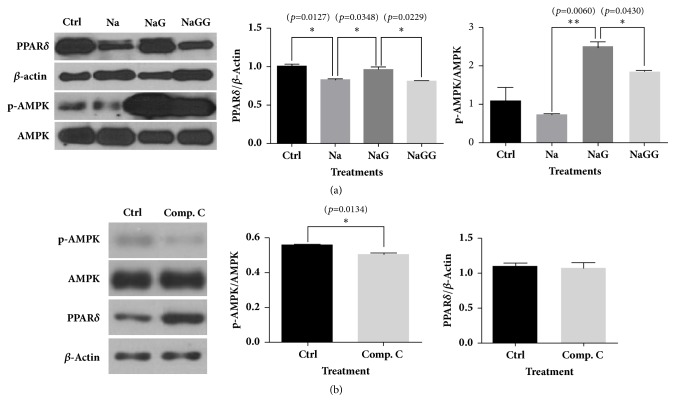
**Western blot analyses for PPAR** **δ** **and AMPK in HEK293 cells treated with NaCl, 6- gingerol, and GSK0660, or treated by compound C.** (a) 6-Gingerol normalized the abnormal expressions of PPAR*δ* and AMPK in HEK293 cells treated with NaCl. In addition, the effects of 6-gingerol were reversed by PPAR*δ* antagonist. (b) The AMPK antagonist, compound C, did not affect the protein level of PPAR in HEK293 cells. The results are expressed as means ± SEM (n=3). Values were statistically analyzed by unpaired* t*-test. All experiments were repeated three or more times. The statistical significance between the two experimental groups was marked as *∗ p *< 0.05 and *∗∗ p *< 0.01. Meaning of indications: Ctrl means untreated normal control group, Na means NaCl treated group, NaG means NaCl and 6-gingerol treated group, and NaGG means NaCl, 6-gingerol, and GSK0660 treated group.

The decrease in protein expression of PPAR*δ* and p-AMPK induced by NaCl treatment was restored by treatment with 6-gingerol; however, the enhancement induced by 6-gingerol was blocked by the PPAR*δ* antagonist, GSK0660 (Figure 5(a)). An AMPK antagonist, compound C, decreased the phosphorylation of the AMPK protein but did not affect the protein expression of PPAR*δ* in HEK293 cells (Figure 5(b)).

### 3.6. 6-Gingerol Decreased Lipid Accumulation in Differentiated 3T3-L1 Cells, but the Effects Were Offset by a PPAR*δ* Antagonist

**Figure 6:**
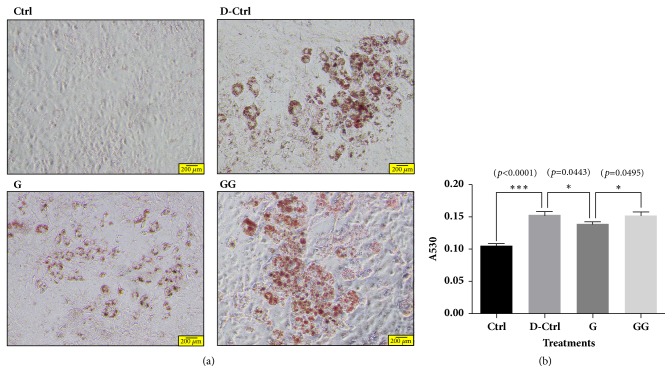
**Oil Red O staining in differentiated 3T3-L1 cells treated with 6-gingerol and GSK0660.** (a, b) 6-Gingerol decreased the lipid accumulation in differentiated 3T3-L1 cells compared to differentiation control. In addition, the effect of 6-gingerol was revered by PPAR*δ* antagonist. Magnification is 200 times. The results are expressed as means ± SEM (n=3). Values were statistically analyzed by unpaired* t*-test. All experiments were repeated three or more times. The statistical significance between the two experimental groups was marked as *∗ p *< 0.05 and *∗∗∗ p *< 0.001. Meaning of indications: Ctrl means untreated normal control group, D-Ctrl means differentiation control group without treatment, G means 6-gingerol treated group during differentiation, and GG means 6-gingerol and GSK0660 treated group during differentiation.

Oil Red O staining of 3T3-L1 cells differentiated in the presence of 6-gingerol and GSK0660 demonstrated that lipid accumulation was decreased by 6-gingerol treatment and that this decrease was reversed by the PPAR*δ* antagonist, GSK0660 (Figure 6).

### 3.7. 6-Gingerol Treatment Increased PPAR*δ*, p-AMPK, and PGC-1*α* Protein Levels in Differentiated 3T3-L1 Cells in a PPAR*δ*-Dependent Manner and Elevated OCRs in Both Undifferentiated and Differentiated 3T3-L1 Cells

**Figure 7:**
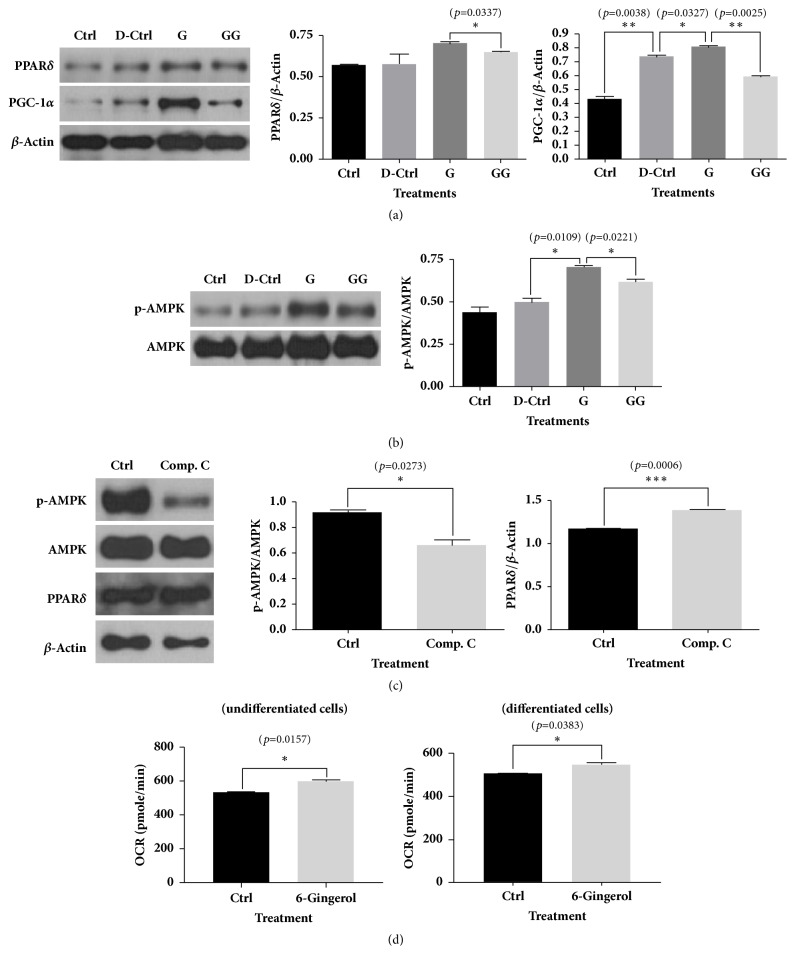
**Western blot analyses for PPAR** **δ** **, AMPK, PGC-1** **α** **in differentiated 3T3-L1 cells treated with 6-gingerol and GSK0660; western blot analyses for PPAR** **δ** **and AMPK in undifferentiated 3T3-L1 cells treated with compound C; the estimation of OCRs in undifferentiated and differentiated 3T3-L1 cells.** (a, b) 6-Gingerol increased the levels for PPAR*δ*, AMPK, and PGC-1*α* compared to differentiation control in differentiated 3T3-L1 cells; however, the effects of 6-gingerol were reversed by PPAR*δ* antagonist. (c) The AMPK antagonist, compound C, did not affect the protein level of PPAR in 3T3-L1 cells. (d) The oxygen consumption rates (OCR) were elevated in both undifferentiated and differentiated 3T3-L1 cells. The results are expressed as means ± SEM (n=3). Values were statistically analyzed by unpaired* t*-test. All experiments were repeated three or more times. The statistical significance between the two experimental groups was marked as *∗ p *< 0.05, *∗∗ p *< 0.01, and *∗∗∗ p *< 0.001. Meaning of indications: Ctrl means untreated normal control group, D-Ctrl means differentiation control group without treatment, G means 6-gingerol treated group during differentiation, and GG means 6-gingerol and GSK0660 treated group during differentiation, Comp. C means AMPK antagonist, compound C.

We confirmed the inhibitory effect of 6-gingerol on lipid accumulation in differentiated 3T3-L1 cells, and to examine the antilipid accumulation mechanism of 6-gingerol, we estimated the protein expression of PPAR*δ*, p-AMPK, and PGC-1*α* in differentiated 3T3-L1 cells. Treatment of differentiated 3T3-L1 cells with 6-gingerol increased the protein expression of PPAR*δ*, p-AMPK, and PGC-1*α* compared with the levels in differentiated control cells. However, the effect of 6-gingerol was offset by the PPAR*δ* antagonist (Figures 7(a) and 7(b)). The AMPK antagonist, compound C, decreased the protein expression of p-AMPK but did not alter the protein expression of PPAR*δ* in 3T3-L1 preadipocytes (Figure 7(c)). Catabolic metabolism of ATP (adenosine triphosphate) is ultimately achieved by the electron transport chain using oxygen as an electron acceptor. Therefore, OCR reflects the extent of catabolic metabolism in cells. The OCR was increased by 6-gingerol treatment in both undifferentiated and differentiated 3T3-L1 cells (Figure 7(d)).

## 4. Discussion

Blood pressure is directly modulated by vascular constriction and relaxation; from this perspective, the role of eNOS is essential in blood pressure regulation. In other words, eNOS lowers blood pressure via vascular relaxation under hypertensive conditions, and the expression of eNOS was shown to be positively regulated by AMPK, PGC-1*α*, and PPAR*δ* in several studies [20–24]. In addition, PPAR*δ* modulated the expression of AMPK and PGC-1*α* as an upstream regulator in hepatocytes and adipocytes under hyperlipidemic conditions [25, 26]. In contrast to eNOS, AT1R, when activated by angiotensin II and atherosclerosis-inducing factors such as TNF*α* and VCAM1 causes hypertension through vasoconstriction [16, 17, 27]. Additionally, in a previous study, hypertensive conditions induced the elevation of AT1R mRNA levels in HUVECs [28]. In our study, 6-gingerol elevated the protein level of p-eNOS in HUVECs. In contrast, 6-gingerol lowered the expression of TNF*α*, VCAM1, and AT1R. From the results in HUVECs, we supposed that 6-gingerol directly alleviated hypertension through the stimulation of vasorelaxation and inhibition of vasoconstriction; in addition, it partially ameliorated hypertension through the downregulation of inflammation and atherosclerosis. In addition to VCAM1, MCP-1 is known to be one of the major chemokines inducing the development of atherosclerosis. [29]. The RT-PCR results in CPAE cells are supported by the significance of the HUVEC data; abnormally expressed PPAR*δ* and MCP-1, which were induced by cholesterol, were normalized with 6-gingerol treatment (additional Figure (available here)). Moreover, the effect of 6-gingerol was posited to be mediated through the sequential regulation of PPAR*δ*-AMPK.

In addition to vascular vessels, the kidney is closely involved in the regulation of blood pressure, and studies on the function of the kidney in blood pressure regulation have reported the following: the ENaC in the distal nephron of the kidney consists of three subunits (*α*, *β*, and *γ*) forming a channel for sodium ion reabsorption. Thus, the kidney modulates blood pressure via the reabsorption of sodium ions through ENaC in a state of low blood pressure. Furthermore, the increased expression of ENaC in the kidney is involved in the development of hypertension [30]; another study has also supported this via investigation of the effect of AMPK on ENaC. Namely, AMPK inhibits ENaC function via the promotion of the interaction between ENaC and ubiquitin ligase Nedd4-2 in HEK293 cells [31]. It is generally known that hypertension induced by high-salt intake originates in increased sodium retention and plasma volume [32, 33].

Moreover, one of the leading causes of high-salt diet-induced hypertension is the elevation of ENaC mRNA expression [34, 35]. In Sprague Dawley (SD) rats fed with a high-salt diet, the expression of p- AMPK was lower than that in the normal diet group but was increased by AICAR [19]. In addition, the activation of AMPK by metformin reduced the increased protein expression of ENaC induced by high-salt conditions in HUVECs [36]. These studies suggest that AMPK is central to the regulation of ENaC expression, and high-salt conditions increase ENaC via the inhibition of AMPK.

In our study, treatment with high sodium chloride concentrations increased the protein expression of ENaC in HEK293 cells; conversely, PPAR*δ* and p-AMPK protein levels decreased. These results are consistent with those of other studies regarding high-salt diet-induced hypertension. Moreover, based on our results, 6-gingerol normalized ENaC, PPAR*δ*, and p-AMPK protein levels in HEK293 cells treated with high sodium chloride, and PPAR*δ* was a more effective upstream regulator than AMPK with respect to the expression of ENaC. Therefore, the normalization of ENaC by 6-gingerol in HEK293 cells treated with high sodium chloride may occur sequentially through the PPAR*δ*-AMPK pathway.

In addition, recent studies strongly suggest the corelation between renal inflammation and hypertension [37, 38]. In this study, the mRNA level of TNF*α* elevated by NaCl was decreased with 6- gingerol treatment, and the regulation of TNF*α* by 6-gingerol was PPAR*δ*-dependent. Therefore, we posit that the decreased inflammation observed after 6-gingerol treatment of kidney cells is one of the significant factors leading to normalization of hypertensive conditions.

Obesity is an essential factor in the induction of hypertension; that is, weight loss caused by exercise and other treatments improves the symptoms of hypertension [39–41]. According to the WHO (World Health Organization) definition [42], obesity is a body state in which abnormally and excessively accumulated fat impairs health. In addition, the normal secretion of adipokines in adipose tissue is disturbed by an obese state, and the abnormal secretion of adipokines may induce hypertension through endothelial dysfunction [43, 44]. Therefore, the inhibition of lipid accumulation in adipose tissue and cells directly lowers elevated blood pressure. Lipid accumulation in adipose tissue can be decreased by the stimulation of the oxidative phosphorylation of fatty acids, and biomarkers such as PPAR*δ*, AMPK, and PGC-1*α* are involved predominantly in fatty acid catabolism in adipose tissue. PPAR*δ* activation lowers lipid content via the upregulation of genes related to fatty acid oxidation [45, 46]. Similar to PPAR*δ*, AMPK stimulates the beta-oxidation of fatty acids but also inhibits lipolysis via the regulation of hormone-sensitive lipase in adipocytes [47, 48].

PGC-1*α* performs an essential role in the conversion of white adipose tissue to brown adipose tissue and is involved in adaptive thermogenesis under cold exposure conditions [49, 50]. Furthermore, PGC-1*α* has an indispensable role in fatty acid oxidation in adipose tissue and cells and is regulated by AMPK: in mice with fat tissue lacking the PGC-1*α* gene, the expression of genes related to lipid oxidation and thermogenesis are reduced; and in PGC-1*α*-overexpressing 3T3-L1 cells, the mRNA expression of genes involved in fatty acid oxidation is elevated [51–53].

In several reports, 6-gingerol inhibited lipid accumulation in 3T3-L1 cells and exerted hypolipidemic effects in leptin receptor-deficient db/db mice [54–56]. Similar to findings from other studies, 6- gingerol may decrease lipid accumulation in differentiated 3T3-L1 cells through the stimulation of PPAR*δ*, AMPK, and PGC-1*α* involved in fatty acid catabolism in this study. Furthermore, the elevation of the OCR following 6-gingerol treatment of 3T3-L1 cells supports that the lipid-lowering effect of 6-gingerol is mainly attributed to the oxidative phosphorylation of fatty acids. In addition, the mRNA levels for PPAR*γ* and FAS participated in fatty acid synthesis were decreased by 6- gingerol treatment in 3T3-L1 cells and suggest that 6-gingerol additionally can inhibit lipid accumulation via the downregulation of biomarkers related to fatty acid synthesis. Furthermore, the lipid-lowering effect of 6-gingerol in 3T3-L1 cells was dependent on PPAR*δ*. These results suggest that 6-gingerol could exert antiobesity and hypolipidemic effects* in vivo*. Therefore, 6-gingerol may help to lower hypertension via amelioration of hyperlipidemia and obesity.

## 5. Conclusion

**Figure 8:**
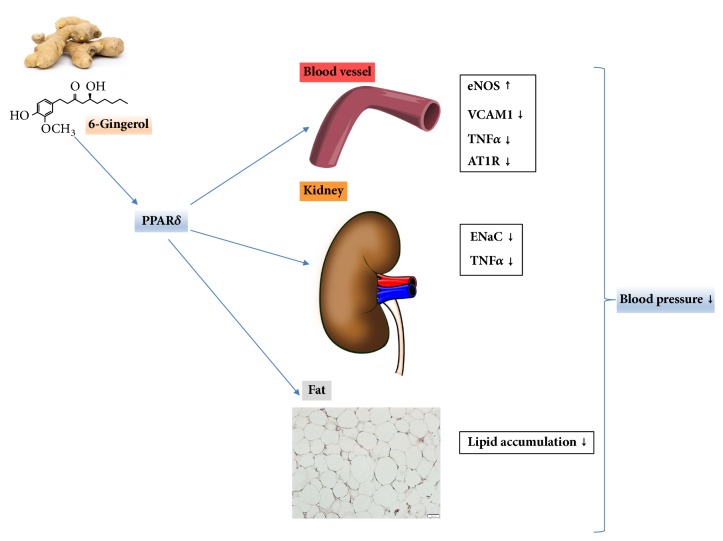
**Hypothetical diagram showing the effects and mechanism of 6-gingerol in the regulation of biomarkers related to hypertension.** The hypothetical mechanism of action of 6-gingerol mediated through PPAR*δ* regulation in hypertension mimicked conditions in vascular endothelial, kidney, and differentiated 3T3-L1 cells.

Consequently, it is suggested that the hypothetical antihypertension functions of 6-gingerol are derived from two routes. The first is the reduction in blood pressure through the amelioration of p- eNOS and AT1R expression in vascular endothelial cells and the downregulation of ENaC and TNF*α* in kidney cells. The second is the amelioration of hypertension via the decrease in lipid accumulation in adipose cells. In addition, all the antihypertensive functions of 6-gingerol may be exerted through PPAR*δ* regulation (Figure 8).

Our study is the first to systemically characterize the antihypertensive mechanism of 6-gingerol using cell-based experiments. However, further investigation of the mechanism for the antihypertensive effects of 6-gingerol will be clarified in future* in vivo *studies.

## Data Availability

The data used to support the findings of this study are available from the corresponding author upon request.

## Supplementary Materials

Supplementary Materials The effects of 6-gingerol on mRNA levels PPAR*δ* and MCP-1 in CPAE cells in pathological condition.
