# Supplementary material for: 6-Gingerol Normalizes the Expression of Biomarkers Related to Hypertension via PPARδ in HUVECs, HEK293, and Differentiated 3T3-L1 Cells
Source: PPAR Res. 2018 Dec 16;2018:6485064. doi: 10.1155/2018/6485064 (PMC6311252; doi:10.1155/2018/6485064)

Additional Figure legend

Additional Figure. The effects of 6-gingerol on mRNA levels PPARδ and MCP-1 in CPAE cells in pathological condition.

6-Gingerol increased the mRNA level of PPARδ decreased by cholesterol in CPAE cells. The 6-gingerol decreased the mRNA level of MCP-1 elevated by cholesterol treatment in CPAE cells. The results are expressed as means ± SEM. Values were statistically analyzed by unpaired *t*-test. All experiments were repeated over three times. ** $p < 0.01$, *** $p < 0.001$. Meaning of indications: Ctrl is an untreated control group, C means cholesterol treated group, CG means cholesterol and 6-gingerol treated group.

Additional Figure

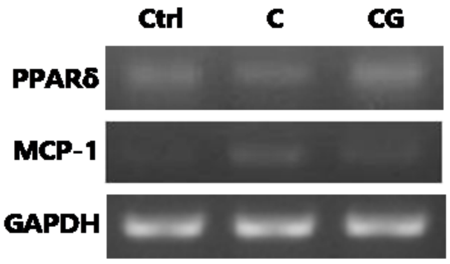

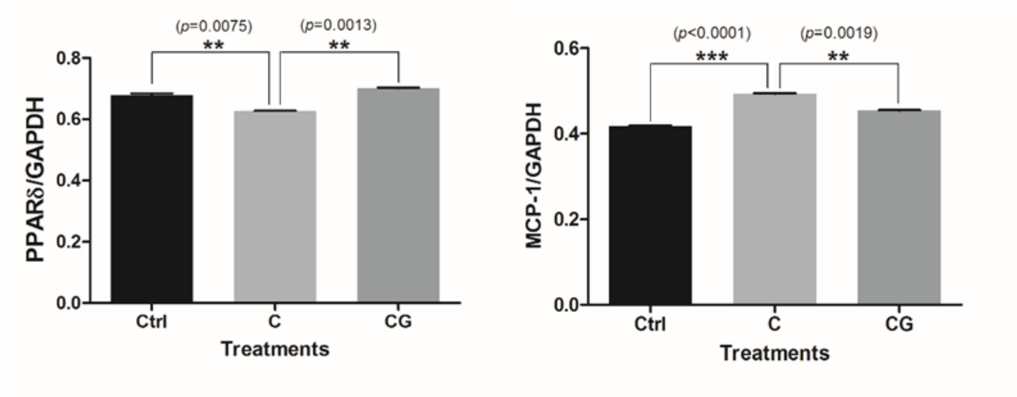

Supplement: Supplementary Materials — The effects of 6-gingerol on mRNA levels PPARδ and MCP-1 in CPAE cells in pathological condition. [file 6485064.f1.pdf]
